# Is there any difference in the therapeutic effects of Levosimendan on advanced HFrEF patients with sinus rhythm or atrial fibrillation?

**DOI:** 10.3389/fcvm.2023.1084300

**Published:** 2023-02-23

**Authors:** Wenyan Wang, Fawen Li, Huihui Huang, Xin Wu, Weixiang Tian, Tao Yu

**Affiliations:** ^1^Department of Heart failure Center, Sichuan Provincial People’s Hospital, Chinese Academy of Sciences Sichuan Translational Medicine Research Hospital, University of Electronic Science and Technology of China, Chengdu, China; ^2^School of Medicine, University of Electronic Science and Technology of China, Sichuan Provincial People’s Hospital, Chengdu, China; ^3^Department of Cardiac Surgery, Sichuan Provincial People’s Hospital, Chinese Academy of Sciences Sichuan Translational Medicine Research Hospital, University of Electronic Science and Technology of China, Chengdu, China

**Keywords:** Levosimendan, heart failure, heart rhythm, atrial fibrillation, ejection fraction

## Abstract

Patients with advanced heart failure have a high incidence of atrial fibrillation (AF) and develop into heart failure with reduced ejection fraction (HFrEF), and require higher doses of inotropes. However, it is uncertain about the differences in the effects of levosimendan in HFrEF patients with sinus rhythm or AF. A total of 63 advanced HFrEF subjects (ejection fraction < 40%) were divided into sinus rhythm (SR, *n* = 34) and atrial fibrillation (AF, *n* = 29) cohorts. All patients received six cycles of intermittent repeated levosimendan infusion. After 3 months of treatment, B-type natriuretic peptide (BNP), estimated glomerular filtration rate, resting heart rate (rHR), creatinine, left ventricle ejection fraction (LVEF), left ventricular end diastolic diameter and blood pressure body weight, NYHA classification were measured. After completing the course of treatment, LVEF, BNP, and rHR were significantly decreased (*p* < 0.0.5), and no significant differences between the two groups were observed (*p* > 0.05). The NYHA classification improved in the SR group but not in the AF group. There was no significant difference between patients with different rHRs (≤70 bpm vs. >70 bpm) in the SR group (*p* > 0.05) or in the AF group (rHR ≤ 90 bpm vs. rHR >90 bpm) (*p* > 0.05). This study showed no difference in the therapeutic effect of intermittent repeated levosimendan infusion on advanced HFrEF with different heart rhythms (SR or AF); Advanced HFrEF patients receive levosimendan treatment without taking the inference of heart rhythm.

## Introduction

Patients with advanced decompensated heart failure who are connected to heart transplantation (Hx) or ventricular assist device support (VAD) can revive intermittent hemodynamic relief from Inotropes (1). However, only a small number of patients with advanced heart failure (AdHF) may get VAD or Hx. However, atrial fibrillation (AF) and decreased left atrial function are frequent complications in patients with advanced HFrEF. Notably, when AdHF with AF reaches a late stage of development (HFrEF), it is characterized by decreased ejection fraction because of the loss of left atrial contractility. Most patients will become inotrope-dependent to relieve the symptoms, reduce re-hospitalization, and create a better quality of life (2, 3). The calcium sensitizer levosimendan can bind to saturated cardiac troponin C and stabilize the calcium, which stabilizes and prolongs the binding of troponin C and I and eventually improves the contractility of the failing heart (4). As an inotropic drug with a half-life of 80 h, levosimendan has been successfully utilized in treating advanced HFrEF patients in the clinic, with good clinical effectiveness and a better prognosis (5).

On the other hand, troponin C, one of the myocardial damage indicators, was found to be higher in AF patients than in Sinus rhythm (SR) patients (6). As the cardiomyocytes are damaged, troponin C is degraded from the myocardial fibers, resulting in a decrease in myocardial fibers. Since levosimendan works by binding to troponin C in myocardial fibers, it is true that individuals with advanced HFrEF with AF have lower troponin C levels than those with advanced HFrEF with SR. The effect of levosimendan on advanced HFrEF with AF should be lower than that of advanced HFrEF with SR, as per theory.

However, there is currently no clinical report on the therapeutic effect of levosimendan on advanced HFrEF in AF or SR patients. This study aimed to determine whether there are differences in the impact of intermittent and repeated levosimendan infusions on the cardiac function of advanced HFrEF with AF or SR patients.

## Materials and methods

**Figure 1:**
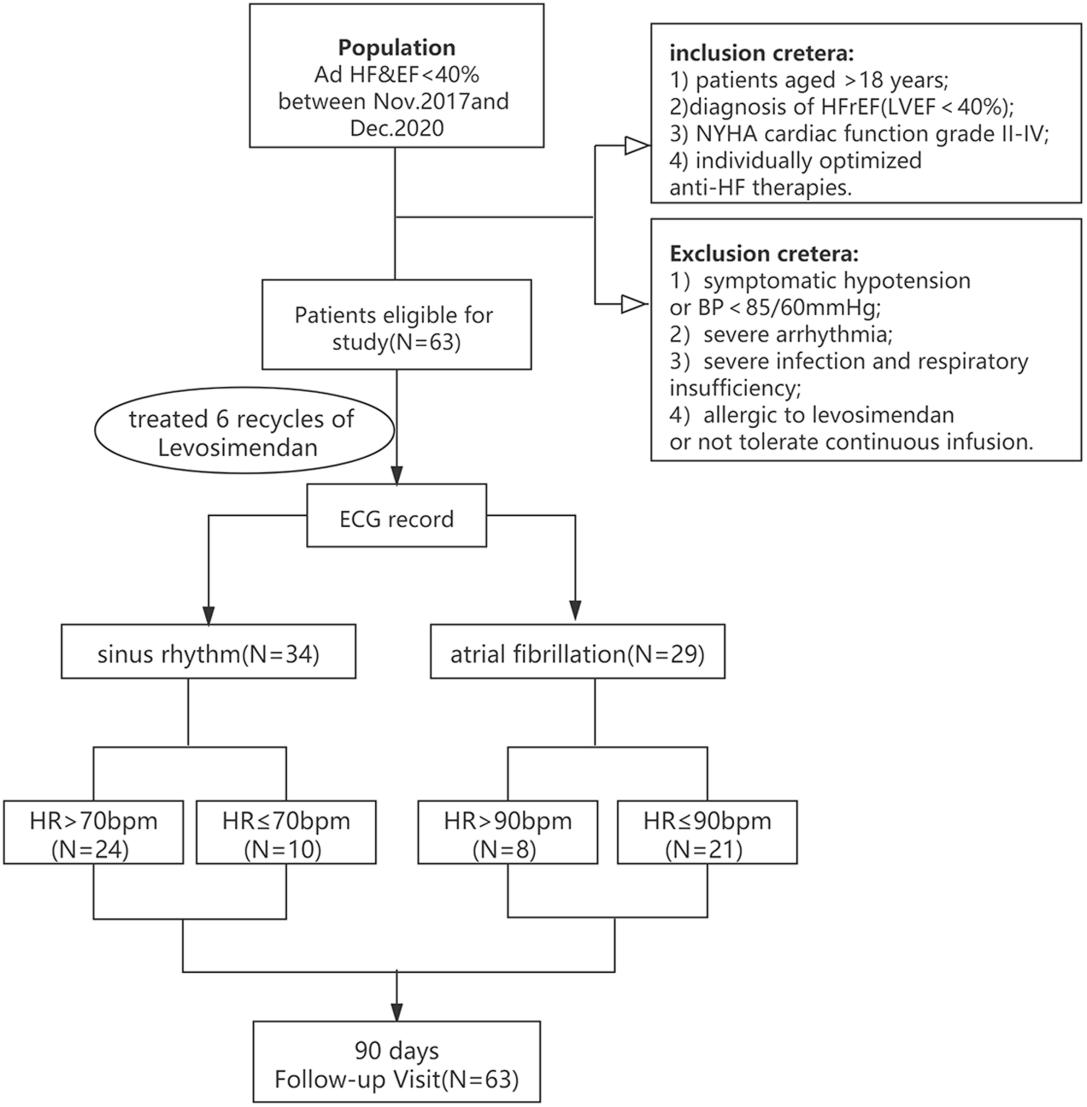
The flowchart for studying the therapeutic effect of levosimendan on advanced HFrEF patients with sinus rhythm or atrial fibrillation.

This was a single-center, retrospective cohort study. The subjects comprised 63 patients with advanced heart failure in our Heart Failure Center from Nov 2017 to Dec 2020. Inclusion criteria: Left ventricle ejection fraction (LVEF) of <40% (measured with Simpson of Doppler Echocardiography); the definition of advanced heart failure with reduced ejection fraction (HFrEF, LVEF<40%). Exclusion criteria: (1) Symptomatic hypotension or blood pressure (BP) <85/60 mmHg; (2) Severe arrhythmia; (3) Severe infection and respiratory diseases; (4) Allergy to levosimendan or unable to tolerate long-term intravenous pumping treatment. The Estimated glomerular filtration rate (eGFR) is calculated following formula: male, Ccr = (140-age)*weight (kg)*1.23/Scr (μmol/L) (Ccr, Creatinine Clearance Rate; Scr, Serum creatinine; female); Ccr = (140-age)*weight (kg)*1.03/Scr (μmol/L). AF atrial fibrillation group (*n* = 29) based on ECG heart rhythm data. Two groups of patients with advanced heart failure received conventional treatment recommended by the guidelines, including diuretics, renin-angiotensin system (RAS) blockers, angiotensin receptor-neprilysin inhibitor (ARNI), aldosterone receptor antagonists, β-receptor blockers, digoxin, etc. Levosimendan was administered in accordance with our center’s approved treatment protocol (7) (the total dose was 12.5 mg, 0.05 ~ 0.2 μg kg^−1^ min^−1^, intravenous 24–48 h, repeated infusion every 2–4 weeks for 3 months). Levosimendan infusion was started at a rate of 0.1 μg kg^−1^·min^−1^ during the first hour and increased to 0.2 μg·kg^−1^·min^−1^ after that if well tolerated in the case of low systolic blood pressure (SBP) (<90 mmHg). Without applying a beginning bolus, levosimendan was infused *via* a 24 h lasting infusion (0.1 ug/kg/min) while being monitored for hemodynamic effects in an intermittent care scenario. We measured the levels of B-type natriuretic peptide (BNP) before and 3 days after the infusion. The patients will repeat the blood test with a total observation time of 6 months to assess the blood pressure, resting heart rate, body weight, NYHA classification, Creatinine (Crea), eGFR, LVEF, left ventricular end diastolic diameter (LVEDD) and BNP. Then proceed to investigate the differences between the two groups (as shown in Figure 1).

### Statistical analysis

Data are presented as (mean values ±standard deviations) for continuous variables and numbers (percentage) for categorical variables. The Student t-test and ANOVA were used for bivariate comparisons of ordinarily distributed continuous variables. The Kruskal-Wallis rank sum test was used for non-normally distributed continuous variables. *p*-values below 0.05 were considered significant.

## Results

### Patient characteristics

Eventually, 63 patients (SR group, n = 34, AF group, n = 29) were included in the study for analysis. For the majority of the demographic information and clinical traits, both groups were well balanced (Table 1).

**Table 1:** Baseline characteristics of AdHF patients with SR and AF (Mean ± SD).

Patient characteristic	SN	AF	*p*
*N*	34	**29**	
AGE	60.912 ± 14.320	65.536 ± 11.852	0.177
Male (%)	70.588	68.966	0.889
Weight (IN)	64.703 ± 15.024	57.944 ± 11.824	0.063
SBP (mmHg)	118.235 ± 20.911	114.690 ± 21.994	0.515
DBP (mmHg)	74.794 ± 15.878	72.241 ± 13.590	0.500
LVEDD (mm)	63.29 ± 10.01	64.60 ± 10.71	0.361
rHR	82.265 ± 18.250	82.138 ± 16.239	0.977
NYHA	3.45 ± 0.62	3.52 ± 0.58	0.687
CREA (μmol/L)	91.929 ± 46.084	100.469 ± 52.021	0.492
BNP (pg/mL)	2,123.000 ± 2,500.334	2,269.167 ± 2,392.024	0.819
eGFR (mL/min. m^2^)	79.639 ± 26.687	73.400 ± 23.838	0.335
Digitalis (%)	50.00	72.41	0.070
beta-blockers (%)	88.24	86.21	0.462
MRA (%)	100.00	100.00	1.000
RAS-inhibitor (%)	100.00	100.00	1.000

SD, standard deviation; SBP, Systolic blood pressure; DBP, diastolic blood pressure; LVEDD, left ventricular end-diastolic dimension; rHR, resting heart rate; NYHA, New York Heart Association; CREA, creatinine; BNP, B-type natriuretic peptide; eGFR, estimated glomerular filtration rate; MRA, mineralocorticoid receptor antagonist; RAS, renin-angiotensin system.

Although the mean age of the AF group was higher than that of the SR group (65.54 ± 11.85 years vs. 60.91 ± 14.32 years, *p* = 0.177), there was no difference between these two groups, neither was the body weight (64.703 ± 15.024 kg vs. 57.944 ± 11.824 kg, *p =* 0.063) nor the blood pressure (SBP:118.235 mmHg ±20.911 vs. 114.690 ± 21.994, *p =* 0.515; DBP: 74.794 mmHg ±15.878 vs. 72.241 mmHg ±13.590, *p =* 0.500), (The AdHF subjects with hypotension that could not tolerate levosimendan for its vasodilation were excluded from the study). NYHA classification (SR 3.45 ± 0.62 *vs* 3.52 ± 0.58 *p* = 0.687). At baseline, the resting heart rate (rHR) was the same (82.27 ± 18.25 bpm vs. 82.14 ± 16.24 bpm, *p =* 0.977). In this study, both groups received standard Guideline-Directed Medical Therapy (GDMT) treatment. The medicine utilization rate of the two groups reached 100% (*p =* 1.00), which was equally balanced, including ACEI/ARB (angiotensin-receptor blocker)/ARNI and spironolactone. There was no significant difference between the two groups for the beta receptor blocker (88.24% vs. 86.21, *p* = 1.00) or digoxin (50% vs. 72.41%, *p* = 0.07).

### Group comparison of patients with SR and AF

After six cycles of intermittent repetitive infusion of levosimendan, BNP, eGFR, LVEF, LVEDD, NYHA classification, rHR, and body weight were assessed and compared with the baseline. The data demonstrated a significant difference in LVEF (27.27 ± 8.25% vs. 44.50 ± 15.02, 26.95 ± 9.33% *vs* 36.55 ± 13.15%; *p* < 0.01), rHR (82.265 ± 18.250 *vs* 71.552 ± 13.991, 82.138 ± 16.239 *vs* 70.655 ± 11.899, *p* < 0.05) and BNP (2,123.000 ng/ml ±2,500.334 *vs* 379.075 ng/ml ±711.863, 2,269.167 ng/ml ± 2,392.024 *vs* 684.542 ng/ml ±1029.799, *p* < 0.01) in SR and AF before or after levosimendan infusion. The LVEDD in SR slightly decreased (63.29 ± 10.01 mm *vs* 60.54 ± 9.91 mm, *p* < 0.05) but no change was seen in AF (64.60 ± 10.71 mm *vs* 61.68 ± 12.06 mm *p* > 0.05). Similarly, cardiac function of the NYHA classification improved in the SR group but not in the AF group. However, there was no significant improvement in eGFR or change in body weight between the two groups.

**Figure 2:**
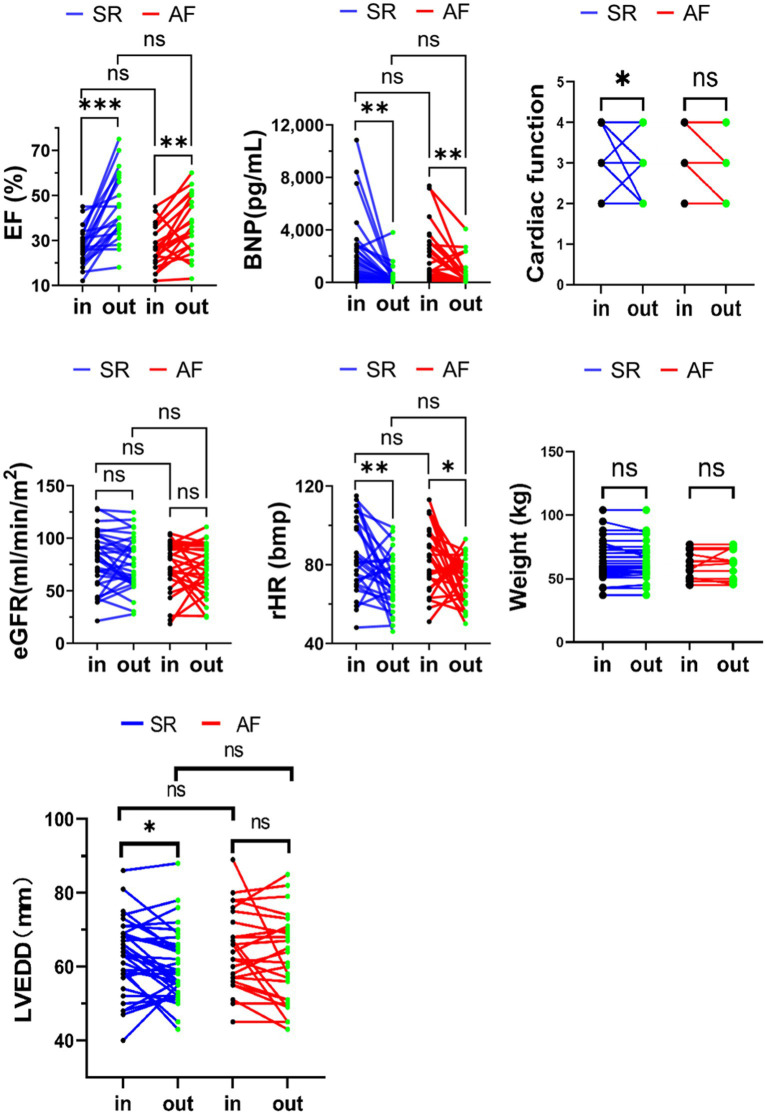
rHR, eGFR, LVEF, body weight, left ventricular end diastolic diameter (LVEDD) cardiac function and BNP variation upon admission and at discharge in advanced HFrEF patients with SR or AF. *p < 0.05; **p < 0.01; ***p < 0.001.

Compared with baseline, for SR and AF, after six cycles of infusion of levosimendan, NYHA classification and LVEDD was significantly improved in SR (*p <* 0.05) but not in AF group; however, there was no significant difference in the changes of BNP, LVEF, LVEDD and NYHA classification between SR and AF (*p* > 0.05). No significant changes were detected in both groups as to eGFR and body weight (*p* > 0.05) (Figure 2).

**Figure 3:**
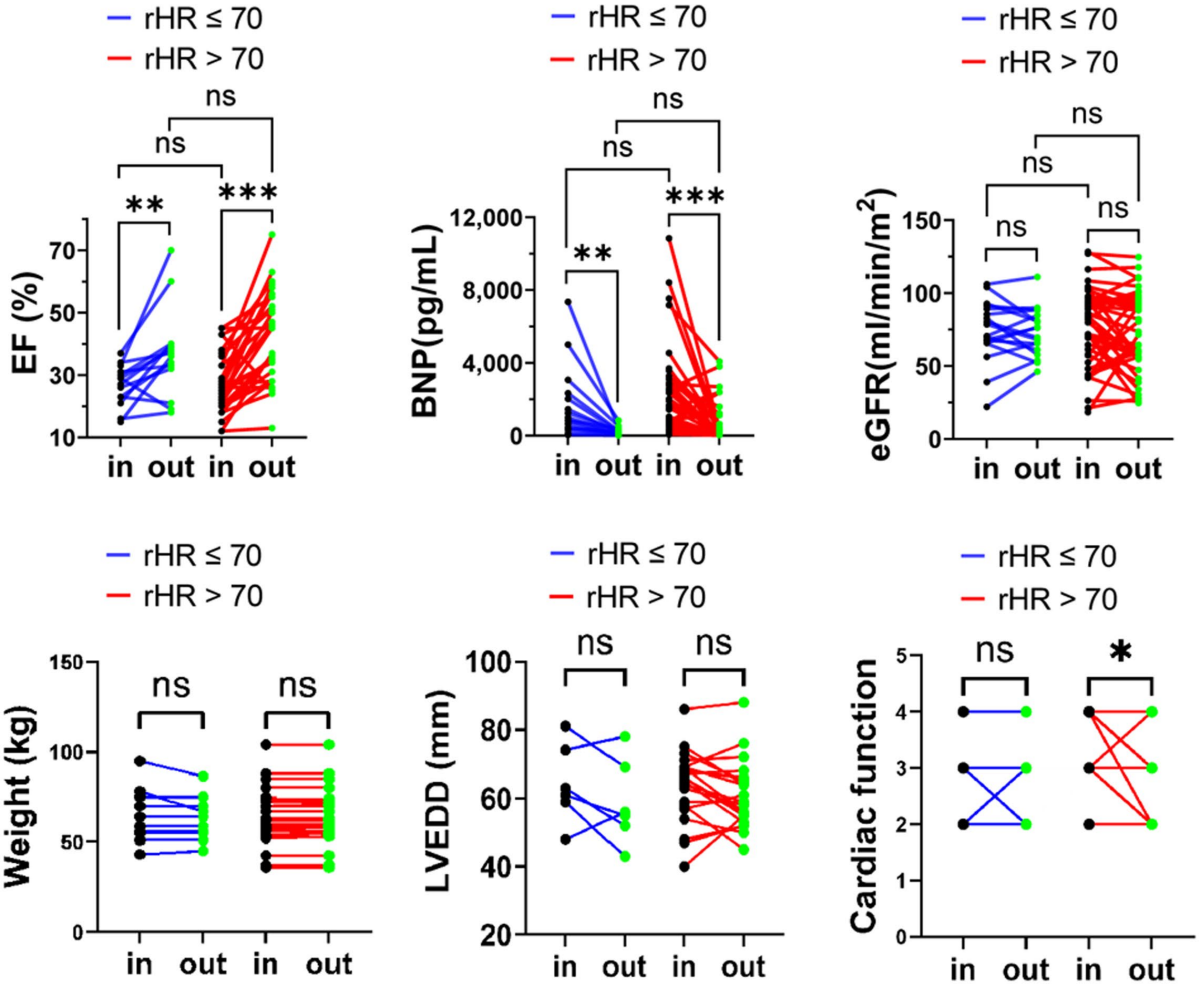
eGFR, LVEF, and BNP body weight, LVEDD, NYHA classification variation upon admission and at discharge in advanced HFrEF patients with rHR ≤ 70 bpm or rHR > 70 bpm. *p < 0.05; **p < 0.01; ***p < 0.001.

Previous studies have proved that the prognosis of advanced HFrEF with sinus rHR <70 bpm was significantly better than that of rHR >70 bpm (5, 7, 8); we used the rHR of 70 bpm in the SR group as a cut-off point to observe the different responses to six cycles of repetitive levosimendan infusion. According to rHR (rHR >70 bpm vs. rHR ≤ 70 bpm), patients with SR were divided into two subgroups, and the changes in BNP, eGFR, LVEF, LVEDD, NYHA classification and body weight were analyzed between these two subgroups. Compared to the baseline, NYHA improved and LVEDD decreased significantly in the rHR ≥ 70 group. However, there were no significant differences in the changes of BNP, LVEF, and eGFR in the two subgroups after levosimendan therapy, including NYHA classification and LVEDD (*p* > 0.05, Figure 3).

**Figure 4:**
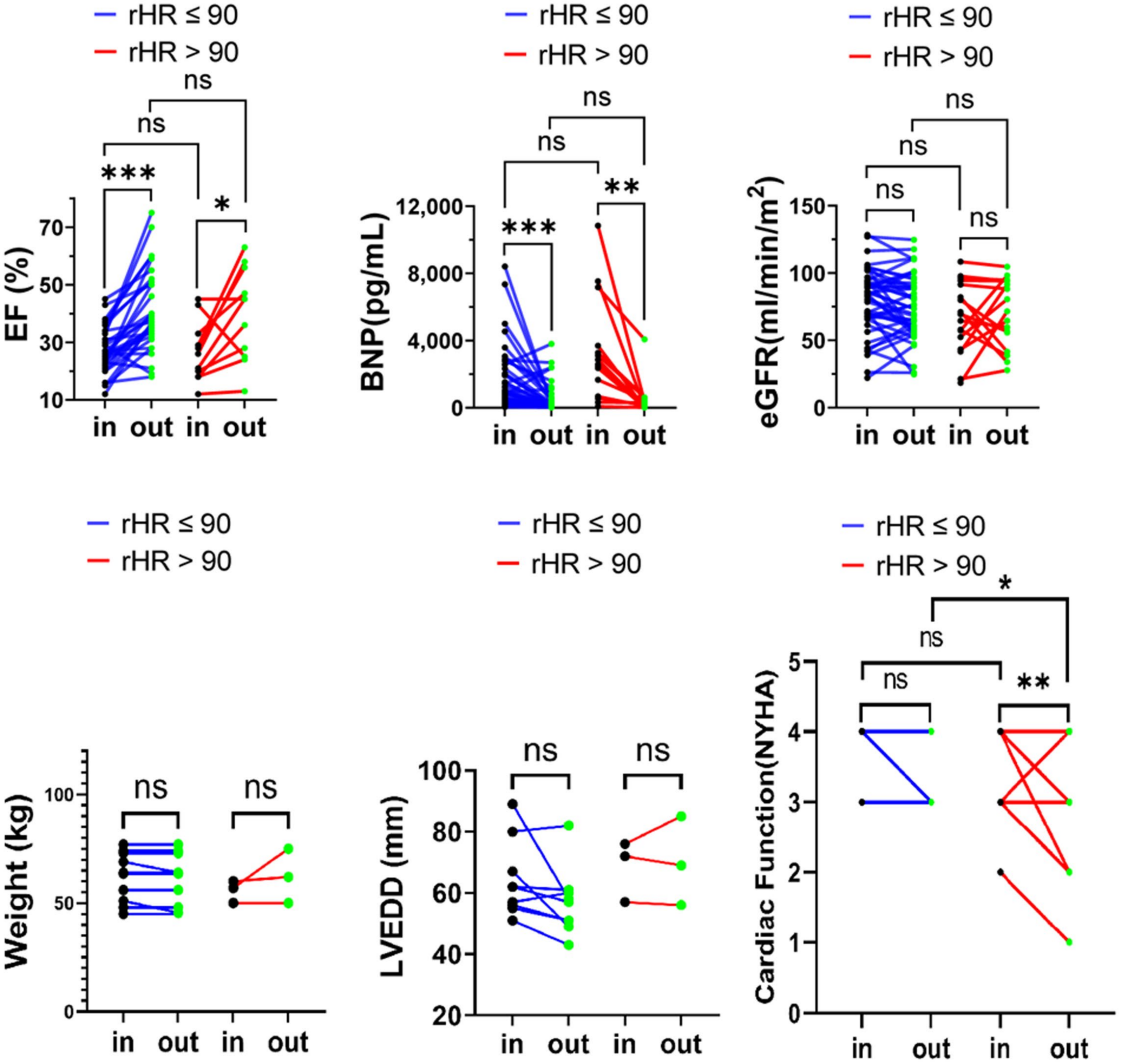
eGFR, LVEF, and BNP body weight, LVEDD, NYHA classification variation upon admission and at discharge in advanced HFrEF patients with rHR ≤ 90 bpm or rHR > 90 bpm. *p < 0.05; **p < 0.01; ***p < 0.001.

Meanwhile, studies have shown that it is appropriate to keep the resting ventricular rate of AF patients with heart failure at around 90 bpm field (2, 9). This maintains cardiac function and counteracts the adverse effects of tachycardia and adverse drug reactions or more pacemaker implantation in patients with strict strategy (9, 10). The cutoff was set at 90 bpm. Similarly, we observed the changes in BNP, eGFR, LVEF, LVEDD, NYHA classification and body weight in the two subgroups (rHR >90 bpm vs. rHR ≤90 bpm). Except for the obvious improvement in NYHA classification in the subgroup with rHR ≤ 90, there were no significant differences (*p* > 0.05) (Figure 4).

## Discussion

Patients who developed advanced heart failure with reduced EF are most likely inotropes-dependent (11). Due to the formation of an active metabolite designated OR-1896 in the systemic and pulmonary hemodynamic field, Levosimendan, a calcium sensitizer, and potassium channel-opener, is valued by specialists in heart failure practitioners for its prolonged duration of effect (1, 5). Repeated and intermittent infusion of levosimendan would be considered as a strategy to improve the quality of life and survival rate for AdHF patients or contribute to mechanical circulatory support or heart transplant (12, 13).

Levosimendan has been proven to increase the ventricular contractility of the failing heart. Ioannis A et al. reported that levosimendan improves LA performance by decreasing E/e’ and E/a while significantly increasing peak expiratory flow (PEF) and LA-contractile strain (14). As AdHF patients are often complicated with AF, will different rhythms lead to different cardiac responses to these inotropes?

Based on combination treatment with digoxin and -blockers for heart failure patients with AF or SR, Can Yontar et al. demonstrated that levosimendan could improve left ventricular hemodynamics. There was no significant difference between the two groups of HF patients. (15). However, an impaired atrial function is associated with atrial fibrillation which might decrease the cardiac response of levosimendan, especially for advanced HFrEF. There is currently insufficient data to say if the therapeutic impact of levosimendan for advanced HFrEF with SR or AF varies between intermittent and recurrent usage. This study was designed to answer this question.

According to the ECG rhythm, the advanced HFrEF patients in this research were split into SR and AF groups. The two groups of heart failure patients received standard GDMT, and heart rate control was relatively stable. According to the RACE II trial field (9), patients with AF could initially receive a lenient heart rate regime; the majority of AdHF patients had AF that was long-lasting or permanent, which reduced the number of patients who had a quicker ventricular rate. Previous studies have proved that the worse the prognosis for heart failure patients with SR, the faster the heart rate. The lower the heart rate, the lower the risk of cardiovascular death and hospitalization (5, 7, 16). However, there is no clear correlation between ventricular rate and prognosis for heart failure patients with AF (17). The baseline of the patient’s characteristics remained unchanged following the administration of previous digoxin or β-blocker treatment, which neutralized the significance of analyzing the effects of levosimendan. There was no significant difference in baseline characteristics between the two groups (18–21).

In this study, changes in LVEF, eGFR, rHR, body weight, LVEDD, NYHA classification and BNP were examined after six cycles of levosimendan infusions for advanced HFrEF, and all patients had a significant increase in LVEF (Figure 2). Intermittent repeated infusion of levosimendan might not only offer continuous positive inotropic support to patients with reduced EF but also give these AdHF patients the opportunity and prerequisites to tolerate a more suitable dosage of GDMT. Levosimendan can help AdHF patients tolerate RAS inhibitors or ARNI, β-blockers, and spironolactone to reverse ventricular remodeling and improve cardiac function (12, 22). The cardiac function and LVEDD in the SR group improved, but not in the AF group. This appears to imply that AdHF with sinus rhythm has an advantage in response to levosimendan. However, it did not show a significant difference when compared to the AF group, so it could not be concluded that AdHF with sinus rhythm had a better response to levosimendan. This also suggested that levosimendan is an inotropic drug, which could improve myocardial contractility. However, in order to achieve positive and valid ventricular reverse remodeling in AdHF patients, it should still rely on neuroendocrine antagonism and require a relatively longer follow-up (23).

This study showed that intermittent repeated levosimendan infusion resulted in a significant decrease in BNP levels, consistent with the results observed in previous studies (12, 24, 25). Levosimendan could promote ventricular emptying and reduce left ventricular end-diastolic pressure and left atrial pressure by increasing ventricular contractility, consequently decreasing BNP (26, 27). Atrial cardiomyocytes can release BNP during atrial fibrillation, except ventricular cells field, even though inotropes have a limited effect on the strain capacity of the left atrium in patients with AF (28). Therefore, BNP levels are usually higher than in patients with SR (29). Hence, the reduction in BNP in AF with levosimendan therapy may be more significant than the reduction in advanced HFrEF with SR (30). However, as per our findings, there was no significant differences in BNP decrease between the AF and SR groups, nor was there any change in baseline BNP level. This further demonstrates that the therapeutic effect of repeated and intermittent levosimendan on AdHF should not be associated with rhythm.

The study did not identify any differences between the SR and AF groups, nor did it demonstrate that intermittent repeated infusions of levosimendan could enhance the GFR of the two groups from the baseline. Previous studies have shown that levosimendan could improve renal function and increase eGFR in patients with acute heart failure (15, 31–33), particularly for patients with low blood pressure, renal insufficiency, or cardiorenal syndrome induced by acute decompensated heart failure, could benefit from maintaining blood volume balance, using levosimendan to increase cardiac output and increase renal perfusion through tube dilation field (34). Additionally, previous studies have discovered that levosimendan treatment improved the eGFR of heart failure patients, with a peak on day three and a return to baseline on day thirty, which suggested levosimendan might have a short-term beneficial effect on renal function of acutely decompensated heart failure patients (35). The subjects in this study were advanced HFrEF patients who lack the significant hemodynamic benefits from intermittent and repeated levosimendan infusion which may fail to advance renal function when relevant indicator parameters are detected, resulting in the minor improvement of renal function after levosimendan treatment in our HF cohort. The preservation of renal function was the same for patients with various heart rhythms.

After levosimendan treatment, compared with baseline, the resting heart rate of all patients decreased (*p* < 0.05), but there was no statistical difference between the AF and SR groups (*p =* 0.94). This might be related to the improvement of cardiac function because the increased output can slow down the heart rate through feedback. The heart rate can be lowered concurrently by using β blockers (and the combination of ivabradine in the SR group) can reduce the heart rate. It has been demonstrated that lowering the resting heart rate will improve the prognosis for heart failure patients with SR (5, 7). This suggests that levosimendan, without any rhythm constraints, might improve the prognosis of this advanced HFrEF.

Previous studies revealed that a high prevalence of atrial high-rate episodes (AHRE) is associated with adverse cardiovascular events (36, 37). In the current study, SR and AF groups were divided into two groups based on different heart rates separately, specifically rHR ≥ 70 bpm vs. <70 bpm for the SR group and rHR ≥ 90 bpm vs. <90 bpm for the AF group. Although after six cycles of treatment, the cardiac function in rHR ≥ 70 group, while rHR < 90 in AF group were improved. There was no significant difference in the therapeutic effect of levosimendan between the heart rate subgroups of SR or AF. The faster the rHR, the more severe the decompensation of heart failure with sinus (38), and the absolute cardiac function benefit from levosimendan would be greater than that with rHR < 70 in SR. A higher heart rate at baseline, on the other hand, was associated with higher all-cause mortality in patients in sinus rhythm but not in AF (39). The effect of resting heart rate on cardiac function in patients with atrial fibrillation is largely unknown, which may lead to contradictory conclusions regarding inotrope intervention. This study found that the cardiac function of the rHR ≥ 90 subgroup was relatively improved, but it could not be concluded that patients with AF and AdHF who have faster rHR would benefit from Levosimendan infusion. Therefore, heart rate has no effect on levosimendan’s effect on cardiac function indicators for SR and AF. It has been reported that AHREs in patients with a history of AF did not significantly increase cardiovascular risk. These findings were consistent with the tendency of our observation results (37).

The results revealed that following treatment with levosimendan, there was no difference in eGFR change rate, BNP decrease percentage, and EF increases between AF and SR groups (*p* > 0.05). As a calcium sensitizer, levosimendan progresses the cardiac contractility independent of intracellular calcium in the flux (26); even in patients with ischemic cardiomyopathy, levosimendan did not increase the incidence of arrhythmia (40). This study also showed that the effects of levosimendan were unaffected by heart rate.

### Limitations

Subjects of AF should be examined with the function or size of the left atrium, which could help identify a link between the various responses to Levosimendan infusion. Additionally, this study did not investigate the data on the occurrence of AHREs in AF groups; using 90 bpm as the cut-off point to observe patients with AF might lead to bias and negative results. Whether the rapid HR of patients with atrial fibrillation will lead to different effects of levosimendan must be further investigated and proved by expanding the samples.

## Conclusion

According to our investigation, patients with advanced HFrEF, whether they had atrial or sinus fibrillation, underwent intermittent repetitive levosimendan infusion for six cycles and three months. The main finding of this study was that there was no difference in the alterations to the heart failure index between the SR and AF groups. In contrast, there was no difference in the secondary outcome between patients with different rHR in AF or SN. It is suggested that rhythm does not affect how levosimendan works to treat advanced HFrEF.

## Data availability statement

The raw data supporting the conclusions of this article will be made available by the authors, without undue reservation.

## Ethics statement

Ethical approval was not provided for this study on human participants because This is a retrospective study. Written informed consent for participation was not required for this study in accordance with the national legislation and the institutional requirements.

## Author contributions

WW and FL collected the epidemiological and clinical data and drafted the manuscript. HH and WT collected and summarized all data. XW draw figures and tables. TY and WW designed the clinical study, analyzed all data, and revised the final manuscript. All authors contributed to the article and approved the submitted version.

## Funding

This work was supported by the Sichuan Science and Technology Program (2020YFQ0060), Sichuan Administration of Traditional Chinese Medicine (2021–112).

## Conflict of interest

The authors declare that the research was conducted in the absence of any commercial or financial relationships that could be construed as a potential conflict of interest.

## Publisher’s note

All claims expressed in this article are solely those of the authors and do not necessarily represent those of their affiliated organizations, or those of the publisher, the editors and the reviewers. Any product that may be evaluated in this article, or claim that may be made by its manufacturer, is not guaranteed or endorsed by the publisher.
